# Surgical and Prosthetic Rehabilitation of Combination Syndrome

**DOI:** 10.1155/2014/186213

**Published:** 2014-01-06

**Authors:** Paolo Carlino, Francesco Pettini, Stefania Cantore, Andrea Ballini, Felice Roberto Grassi, Valentina Pepe

**Affiliations:** Department of Dental Sciences and Surgery, University of Bari “Aldo Moro,” Piazza G. Cesare No. 11, 70124 Bari, Italy

## Abstract

The aim of this report is to analyze the clinical symptoms, ethologic factors, and prosthetic rehabilitation in a case of Combination Syndrome (CS). The treatment of CS can be conventional or surgical, with or without the bone reconstruction of maxilla. The correct prosthetic treatment helps this kind of patients to restore the physiologic occlusion plane to allow a correct masticatory and aesthetic function. Management of this kind of patients can be a challenge for a dental practitioner.

## 1. Introduction

The seventh edition of the *Glossary of Prosthodontic Terms *defines *Combination Syndrome *(CS) as “the characteristic features that occur when an edentulousmaxilla is opposed by natural mandibular anterior teeth, including loss of bone from the anterior portion of the maxillary ridge, overgrowth of the tuberosities, papillary hyperplasia of the hard palate's mucosa, extrusion of the lower anterior teeth, and loss of alveolar bone and ridge height beneath the mandibular removable partial denture bases—also called anterior hyperfunction syndrome” [1, 2].

This matches the findings of Kelly on the pattern of residual ridge resorption as observed in a group of patients completely wearing maxillary dentures opposing distal extension removable partial dentures (RPD).

Saunders noted an associated loss of vertical dimension of occlusion, occlusal plane discrepancy, anterior repositioning of the mandible, poor adaptation of the prostheses, epulis fissuratum, and periodontal changes [3].

Kelly considered the early bone loss in the anterior maxilla to be the key to the other changes and noted that as resorption of the premaxilla progressed, further tissue damage and denture instability followed proportionately [2, 4, 5].

The changes in tissue form and health seen in Combination Syndrome can be attributed to several factors. When mandibular anterior teeth are present, patients tend to favor these teeth functionally because of the ability to produce maximum force. Excessive anterior functional and parafunctional forces, particularly when not counterbalanced posteriorly in excursive movements, constantly overload the anterior ridge to result in alveolar bone resorption [2, 6, 7].

As bone and ridge height are lost anteriorly, tuberosities in the posterior site will often enlarge and grow downward.

One theory suggests that negative pressure within the maxillary denture pulls the tuberosities down as the anterior ridge is driven upward by the anterior occlusion.

The functional load will then direct stress to the mandibular distal extension and cause bony resorption of the posterior mandibular ridge.

The upward tipping movement of the anterior portion of the maxillary denture and the simultaneous downward movement of the posterior portion will decrease antagonistic forces on the mandibular anterior teeth and lead to their supraeruption [6, 8].

Eventually, an occlusal plane discrepancy will occur and the patient may have a loss of vertical dimension of occlusion.

In addition, the chronic stress and movement of the denture will often result in an ill-fitting prosthesis and contribute to the formation of palatal hyperplasia [7].

According to Tolstunov [9], CS can be classified into the following.

### 1.1. Class I

*Maxilla:* completely edentulous alveolar ridge. *Mandible:* Modification 1 (M1): partially edentulous ridge with preserved anterior teeth only. Modification 2 (M2): stable “fixed” full dentition (natural teeth or implant-supported crowns/bridges). Modification 3 (M3): partially edentulous ridge with preserved teeth in anterior and one posterior region.

### 1.2. Class II

*Maxilla:* partially edentulous alveolar ridge with teeth present in both posterior regions, edentulous and atrophic anterior region. *Mandible:* modifications are the same as in Class I (M1, M2, and M3).

### 1.3. Class III

*Maxilla:* partially edentulous alveolar ridge with teeth present in one posterior region only, edentulous and atrophic anterior and one posterior region. *Mandible:* modifications are consistent with Classes I and II (M1, M2, M3A, and M3B) [9–12].

## 2. Case Presentation

We report a case of a 28-year-old female patient who came to our attention referring to suffering from a precocious loss of teeth due to the periodontal disease; for this reason the patient was wearing a removable maxillary partial denture of which she was not satisfied neither functionally nor aesthetically. This device was, in fact, unsettled and inadequate. Aesthetic and phonetic tests showed a vertical dimension deficit, accompanied with an anterior sliding of mandible and with an alteration of occlusal plan.

There were no mucous oral pathologies.

Clinically we noticed the following:

- partial edentulism of the upper maxilla with only the presence of the following dental elements: 1.6, 1.7, and 2.7; furthermore, we relieved a strong premaxilla atrophy and fluctuating crest;
- partial edentulism in the posterior mandible accompanied by extrusion of frontal teeth.

**Figure 1:**
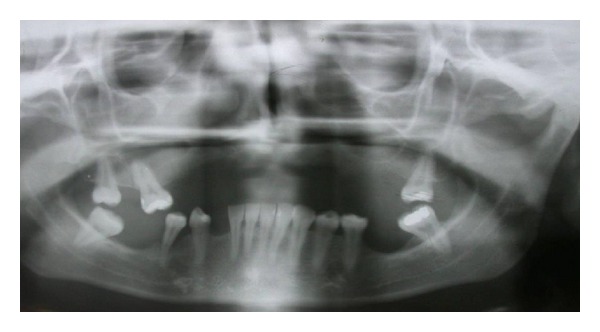
Combination Syndrome (CS) case of partially edentulous maxilla and mandible: unopposed mandibular anterior teeth are supererupted towards the atrophic premaxillary bone. Panoramic radiograph depicting anterior maxillary bone loss and showing the bone remodeling changes of the alveolar process common for CS: resorption of anterior maxilla and posterior mandible. CS classification: Class II, Mod. 3 (II-3);

**Figure 2:**
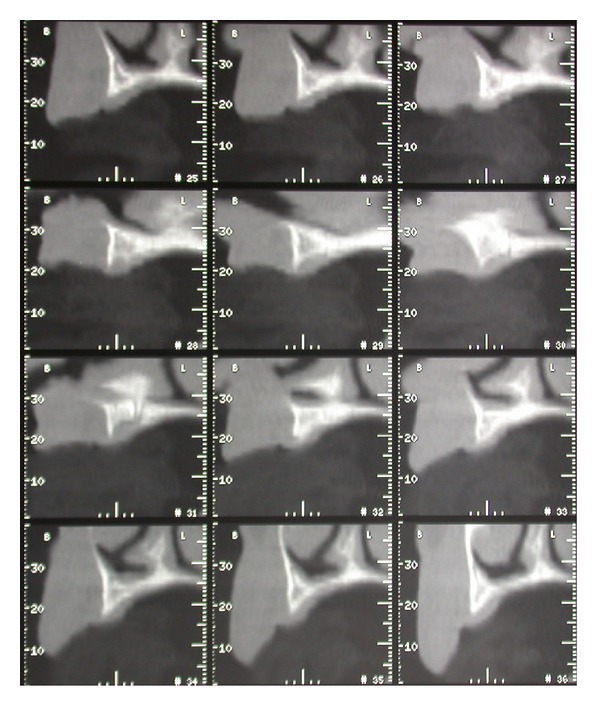
Tomographic images showing severe maxillary resorption to the basal bone.

The radiographic examination showed strong atrophy of the premaxilla with reabsorption of the edentulous crest both horizontal and vertical (Figures 1 and 2).

## 3. Diagnosis

On the basis of Tolstunov classification [9], a clinical and radiographic diagnosis of CS Class II, Mod. 3 (II-3) was made.

## 4. Treatment Plan

We decided for an implant-prosthetic treatment plan of the upper maxilla.

At the initial surgical consultation, the general oral condition and severe bone atrophy were discussed with the patient, and the informed consent was obtained from her.

Prosthetic evaluation has been made first by mounting casts on articulator and then manufacturing new prosthesis that allow the aesthetic and functional rehabilitation of this case report.

The same prostheses have been useful as surgical guides for the reconstruction of the upper maxilla that, in this case, has been done with autologous bone graft obtained from the iliac crest.

**Figure 3:**
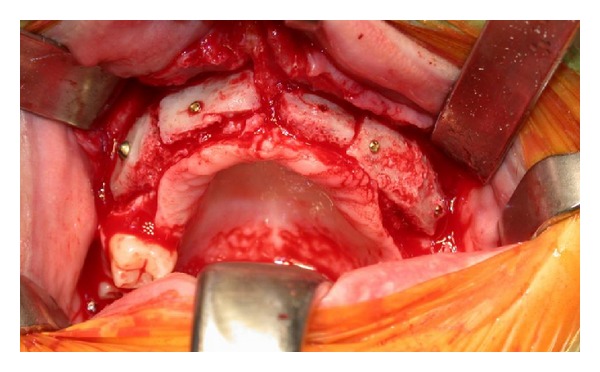
Cancellous bone grafting material, consisting of autogenous bone that was harvested from the iliac crest and placed in the created subantral pocket.

Autologous bone blocks have been shaped to adapt perfectly to the edentulous crest surface and have been fixed with titanium screws (Figure 3).

**Figure 4:**
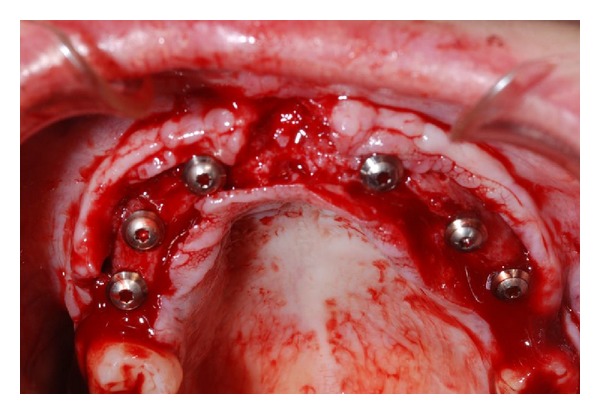
Implant insertion in sectors 1.2, 1.4, 1.6, 2.2, 2.4, and 2.6.

After four months, necessary to the consolidation of the bone graft, 6 Straumann SLActive implants with 4,1 mm of diameter and 10 mm of length have been positioned (Figure 4).

Once the crestal incision was done, the access edge has been unstuck with total thickness; titanium microscrews that maintained the graft have been unscrewed and then implants in sectors 1.2, 1.4, 1.6, 2.2, 2.4, and 2.6 have been positioned using a surgical guide previously realized. At the end, edges were sutured.

During the whole period necessary for implants osseointegration, the patient has used the preceding prosthesis, conveniently spaced out and treated with resilient materials.

After two months, necessary period for implant osseointegration, we proceeded to the prosthetic finalisation.

**Figure 5:**
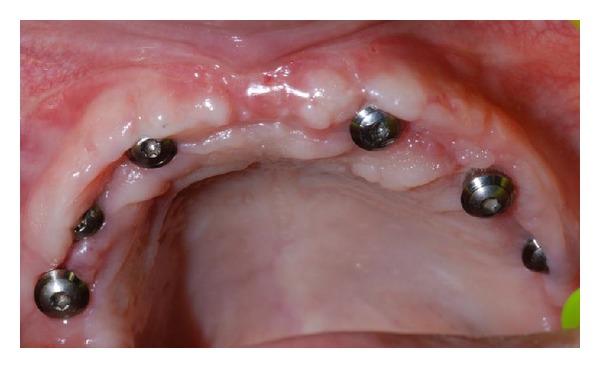
Clinical postoperative photo of the patient after tissue healing.

Firstly, we verify the soft tissues healing (Figure 5), and subsequently an extended impression of the upper maxilla was taken.

On the relative model has been prepared an individualized tray that allowed us to take in a faithful way implants position.

Precision impression has been droped three times: on the first cast (master) the prosthetic finalisation has been executed, on the second model the provisional one has been executed, and the third model has been used for laboratory procedures.

An occlusal basic wax was prepared and this one helped us to obtain the vertical dimension to transfer the correct spatial position of the upper maxillary cast to articulator (using the facial bow) and to test phonetic and preoral tissues support.

The appropriate abutments were selected and the temporary prosthesis was built.

This prosthesis has confirmed the goodness of modifications made to the occlusal pattern and it verified phonetic and aesthetic function of the final prosthesis already tested with the removable prosthesis.

Besides, we controlled the adequate oral hygiene maintenance.

**Figure 6:**
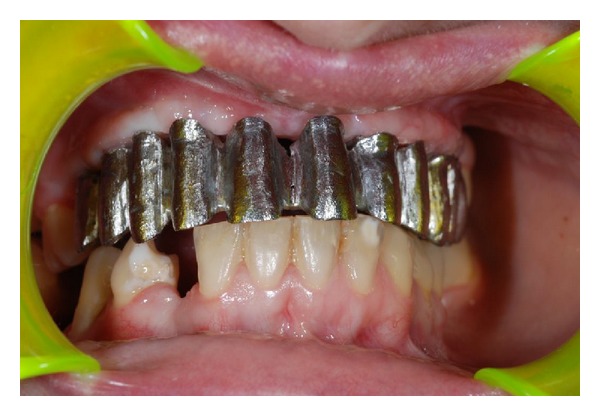
A platinum-palladium-gold (type IV) alloy framework of the mesobar cemented on implant abutments with O-ring male implant attachments for retention of the partial implant overdenture.

After the tray of the metal framework, the laboratories finalized the restoration (Figure 6).

**Figure 7:**
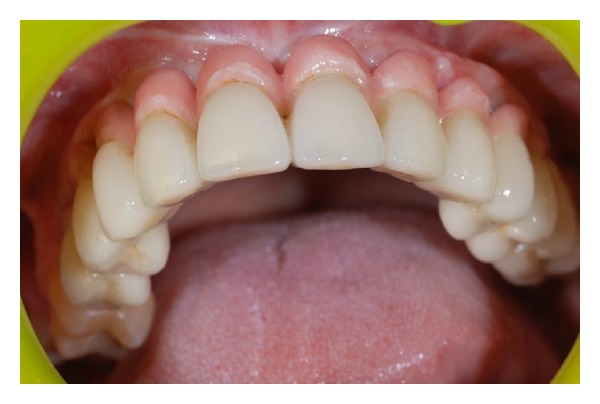
Postcompletion photograph of the patient in the case report wearing the maxillary implant overdenture.

Before the procedure was concluded, the patient received instructions for function and hygiene. Patient's prospective for the improved retention of her upper partial denture was met and she was satisfied with the final result (Figure 7).

We follow up the patient after two weeks and every month during the first year in order to check the soft and hard tissues around implants and the occlusal stability.

## 5. Discussion

Today the CS if often observed in patients wearing complete maxillary denture opposing to complete denture retained to implants by bars or ball-attachments, showing biomechanical similarities between overdenture on implants and distal-extension removable partial dentures supported by mini implants.

Previous to or at the implant surgical stage, the hypertrophy of posterior maxilla and overgrowth of maxillary tuberosities can be corrected with an alveoloplasty and maxillary implants can be placed in a better vertical association. If subantral expansion (sinus lift) is needed, this can also be done with a direct (Tatum) or indirect (Summers) technique.

Implants supported prosthesis can develop higher bite forces compared with traditional prosthesis and this can produce significant biomechanical stress to anterior maxilla [13, 14].

The surgical treatment of CS with maxilla reconstruction restores the correct relationship between skeletal basis and allows the insertion of implants in a prosthetically oriented way.

It is possible to stabilize the maxillary prosthesis, the occlusal vertical dimension, and the occlusal plane.

A careful followup of CS patient is always required [9, 15].
